# Supplementary material for: Effect of Local Adjuvants Following Curettage of Benign and Intermediate Tumours of Bone: A Systematic Review of the Literature
Source: Cancers (Basel). 2023 Aug 25;15(17):4258. doi: 10.3390/cancers15174258 (PMC10487159; doi:10.3390/cancers15174258)
Supplement: Supplementary file 1 [file cancers-15-04258-s001.zip › cancers-2532605-supplementary.pdf]

Supplementary Materials

Manuscript Title: Effect of local adjuvants following curettage of benign and intermediate tumours of bone. A systematic review of literature.

Content

Supplementary Table S1. PubMed search terms.

Supplementary Table S2. Treatment combination types identified within the systematic review.

Supplementary Table S1. PubMed search terms.

adjuvant treatment AND curettage AND benign bone tumour	("adjuvancy"[All Fields] OR "adjuvanted"[All Fields] OR "adjuvanting"[All Fields] OR "adjuvants"[All Fields] OR "adjuvants pharmaceutic"[Pharmacological Action] OR "adjuvants immunologic"[Pharmacological Action] OR "adjuvants, pharmaceutic"[MeSH Terms] OR ("adjuvants"[All Fields] AND "pharmaceutic"[All Fields]) OR "pharmaceutic adjuvants"[All Fields] OR "adjuvant"[All Fields] OR "adjuvants, immunologic"[MeSH Terms] OR ("adjuvants"[All Fields] AND "immunologic"[All Fields]) OR "immunologic adjuvants"[All Fields] OR "adjuvated"[All Fields] OR "adjuvation"[All Fields] OR "adjuvent"[All Fields]) AND ("therapeutics"[MeSH Terms] OR "therapeutics"[All Fields] OR "treatments"[All Fields] OR "therapy"[MeSH Subheading] OR "therapy"[All Fields] OR "treatment"[All Fields] OR "treatment s"[All Fields]) AND ("curettage"[MeSH Terms] OR "curettage"[All Fields] OR "curettages"[All Fields] OR "curettaged"[All Fields]) AND (("benign"[All Fields] OR "benignancies"[All Fields] OR "benignancy"[All Fields] OR "benignant"[All Fields] OR "benigne"[All Fields] OR "benignity"[All Fields] OR "benigns"[All Fields]) AND ("bone neoplasms"[MeSH Terms] OR ("bone"[All Fields] AND "neoplasms"[All Fields]) OR "bone neoplasms"[All Fields] OR ("bone"[All Fields] AND "tumour"[All Fields]) OR "bone tumour"[All Fields])) AND 1994/01/01:2019/12/31[Date - Publication]
adjuvant treatment AND curettage AND intermediate bone tumour	("adjuvancy"[All Fields] OR "adjuvanted"[All Fields] OR "adjuvanting"[All Fields] OR "adjuvants"[All Fields] OR "adjuvants pharmaceutic"[Pharmacological Action] OR "adjuvants immunologic"[Pharmacological Action] OR "adjuvants, pharmaceutic"[MeSH Terms] OR ("adjuvants"[All Fields] AND "pharmaceutic"[All Fields]) OR "pharmaceutic adjuvants"[All Fields] OR "adjuvant"[All Fields] OR "adjuvants, immunologic"[MeSH Terms] OR ("adjuvants"[All Fields] AND "immunologic"[All Fields]) OR "immunologic adjuvants"[All Fields] OR "adjuvated"[All Fields] OR "adjuvation"[All Fields] OR "adjuvent"[All Fields]) AND ("therapeutics"[MeSH Terms] OR

	"therapeutics"[All Fields] OR "treatments"[All Fields] OR "therapy"[MeSH Subheading] OR "therapy"[All Fields] OR "treatment"[All Fields] OR "treatment s"[All Fields]) AND ("curettage"[MeSH Terms] OR "curettage"[All Fields] OR "curettages"[All Fields] OR "curettaged"[All Fields]) AND (("intermediate"[All Fields] OR "intermediated"[All Fields] OR "intermediately"[All Fields] OR "intermediates"[All Fields]) AND ("bone neoplasms"[MeSH Terms] OR ("bone"[All Fields] AND "neoplasms"[All Fields]) OR "bone neoplasms"[All Fields] OR ("bone"[All Fields] AND "tumour"[All Fields]) OR "bone tumour"[All Fields])) AND 1994/01/01:2019/12/31[Date - Publication]
adjuvants AND curettage AND benign bone tumour	("adjuvancy"[All Fields] OR "adjuvanted"[All Fields] OR "adjuvanting"[All Fields] OR "adjuvants"[All Fields] OR "adjuvants pharmaceutic"[Pharmacological Action] OR "adjuvants immunologic"[Pharmacological Action] OR "adjuvants, pharmaceutic"[MeSH Terms] OR ("adjuvants"[All Fields] AND "pharmaceutic"[All Fields]) OR "pharmaceutic adjuvants"[All Fields] OR "adjuvant"[All Fields] OR "adjuvants, immunologic"[MeSH Terms] OR ("adjuvants"[All Fields] AND "immunologic"[All Fields]) OR "immunologic adjuvants"[All Fields] OR "adjuvated"[All Fields] OR "adjuvation"[All Fields] OR "adjuvent"[All Fields]) AND ("curettage"[MeSH Terms] OR "curettage"[All Fields] OR "curettages"[All Fields] OR "curettaged"[All Fields]) AND (("benign"[All Fields] OR "benignancies"[All Fields] OR "benignancy"[All Fields] OR "benignant"[All Fields] OR "benigne"[All Fields] OR "benignity"[All Fields] OR "benigns"[All Fields]) AND ("bone neoplasms"[MeSH Terms] OR ("bone"[All Fields] AND "neoplasms"[All Fields]) OR "bone neoplasms"[All Fields] OR ("bone"[All Fields] AND "tumour"[All Fields]) OR "bone tumour"[All Fields])) AND 1994/01/01:2019/12/31[Date - Publication]
adjuvants AND curettage AND intermediate bone tumour	("adjuvancy"[All Fields] OR "adjuvanted"[All Fields] OR "adjuvanting"[All Fields] OR "adjuvants"[All Fields] OR "adjuvants pharmaceutic"[Pharmacological Action] OR "adjuvants immunologic"[Pharmacological Action] OR "adjuvants,

	pharmaceutic"[MeSH Terms] OR ("adjuvants"[All Fields] AND "pharmaceutic"[All Fields]) OR "pharmaceutic adjuvants"[All Fields] OR "adjuvant"[All Fields] OR "adjuvants, immunologic"[MeSH Terms] OR ("adjuvants"[All Fields] AND "immunologic"[All Fields]) OR "immunologic adjuvants"[All Fields] OR "adjuvated"[All Fields] OR "adjuvation"[All Fields] OR "adjuvent"[All Fields]) AND ("curettage"[MeSH Terms] OR "curettage"[All Fields] OR "curettages"[All Fields] OR "curettaged"[All Fields]) AND (("intermediate"[All Fields] OR "intermediated"[All Fields] OR "intermediately"[All Fields] OR "intermediates"[All Fields]) AND ("bone neoplasms"[MeSH Terms] OR ("bone"[All Fields] AND "neoplasms"[All Fields]) OR "bone neoplasms"[All Fields] OR ("bone"[All Fields] AND "tumour"[All Fields]) OR "bone tumour"[All Fields])) AND 1994/01/01:2019/12/31[Date - Publication]
curettage AND adjuvant treatment AND benign bone tumour	("curettage"[MeSH Terms] OR "curettage"[All Fields] OR "curettages"[All Fields] OR "curettaged"[All Fields]) AND (("adjuvancy"[All Fields] OR "adjuvanted"[All Fields] OR "adjuvanting"[All Fields] OR "adjuvants"[All Fields] OR "adjuvants pharmaceutic"[Pharmacological Action] OR "adjuvants immunologic"[Pharmacological Action] OR "adjuvants, pharmaceutic"[MeSH Terms] OR ("adjuvants"[All Fields] AND "pharmaceutic"[All Fields]) OR "pharmaceutic adjuvants"[All Fields] OR "adjuvant"[All Fields] OR "adjuvants, immunologic"[MeSH Terms] OR ("adjuvants"[All Fields] AND "immunologic"[All Fields]) OR "immunologic adjuvants"[All Fields] OR "adjuvated"[All Fields] OR "adjuvation"[All Fields] OR "adjuvent"[All Fields]) AND ("therapeutics"[MeSH Terms] OR "therapeutics"[All Fields] OR "treatments"[All Fields] OR "therapy"[MeSH Subheading] OR "therapy"[All Fields] OR "treatment"[All Fields] OR "treatment s"[All Fields])) AND (("benign"[All Fields] OR "benignancies"[All Fields] OR "benignancy"[All Fields] OR "benignant"[All Fields] OR "benigne"[All Fields] OR "benignity"[All Fields] OR "benigns"[All Fields]) AND ("bone neoplasms"[MeSH Terms] OR ("bone"[All Fields] AND "neoplasms"[All Fields]) OR "bone neoplasms"[All Fields] OR ("bone"[All

	Fields] AND "tumour"[All Fields]) OR "bone tumour"[All Fields])) AND 1994/01/01:2019/12/31[Date - Publication]
curettage AND adjuvant treatment AND intermediate bone tumour	("curettage"[MeSH Terms] OR "curettage"[All Fields] OR "curettages"[All Fields] OR "curettaged"[All Fields]) AND (("adjuvancy"[All Fields] OR "adjuvanted"[All Fields] OR "adjuvanting"[All Fields] OR "adjuvants"[All Fields] OR "adjuvants pharmaceutic"[Pharmacological Action] OR "adjuvants immunologic"[Pharmacological Action] OR "adjuvants, pharmaceutic"[MeSH Terms] OR ("adjuvants"[All Fields] AND "pharmaceutic"[All Fields]) OR "pharmaceutic adjuvants"[All Fields] OR "adjuvant"[All Fields] OR "adjuvants, immunologic"[MeSH Terms] OR ("adjuvants"[All Fields] AND "immunologic"[All Fields]) OR "immunologic adjuvants"[All Fields] OR "adjuvated"[All Fields] OR "adjuvation"[All Fields] OR "adjuvent"[All Fields]) AND ("therapeutics"[MeSH Terms] OR "therapeutics"[All Fields] OR "treatments"[All Fields] OR "therapy"[MeSH Subheading] OR "therapy"[All Fields] OR "treatment"[All Fields] OR "treatment s"[All Fields])) AND (("intermediate"[All Fields] OR "intermediated"[All Fields] OR "intermediately"[All Fields] OR "intermediates"[All Fields]) AND ("bone neoplasms"[MeSH Terms] OR ("bone"[All Fields] AND "neoplasms"[All Fields]) OR "bone neoplasms"[All Fields] OR ("bone"[All Fields] AND "tumour"[All Fields]) OR "bone tumour"[All Fields])) AND 1994/01/01:2019/12/31[Date - Publication]

Supplementary Table S2. Treatment combination types identified within the systematic review.

Nr	Surgical Therapy		Adjuvant									Count (n)	Reference
	Curettage	High-Speed Burr	Phenol	Cryo-therapy	Ethanol	Electro-cauterisation	Argon-Plasma Laser	H_2O_2	PMMA	Sodium Hyponitrite	Liquid Zinc Chloride		
1	■											620	[9,10,13,16,19,23,25,28,30,36,38,39,46,47,49,57,59,62]
2	■	■										774	[10,13,16,20,26,33-35,43-45,50,54,58,61,65]
3	■		■									22	[9,40]
4	■	■	■									183	[10,18,35,44,52-54]
5	■		■						■			169	[9,15,23,40]
6	■	■	■						■			131	[21,35,48,60,63]
7	■			■								39	[9,51,66]
8	■	■		■								152	[17,27,33,37,41,43,55]
9	■			■					■			147	[51]
10	■	■		■					■			101	[21,24,29,55]
11	■								■			243	[9,13,15,23,25,57,62]
12	■	■							■			173	[13,16,21,35,45,67]
13	■							■	■			1	[9]
14	■	■						■				2	[20]
15	■	■						■	■			71	[13,20-22]
16	■		■		■							85	[56]
17	■	■	■		■				■			3	[67]
18	■		■		■				■			82	[55]
19	■				■							12	[68]
20	■	■			■							11	[36,68]
21	■				■				■			1	[68]
22	■	■			■				■			37	[48,68]
23	■	■					■					17	[10]
24	■	■					■	■				4	[60]
25	■	■					■	■	■			38	[60]
26	■										■	95	[8]
27	■	■			■	■						31	[32]
28	■	■			■	■			■			1	[32]
29	■	■				■			■			21	[64]
30	■	■	■			■			■			23	[42]
31	■					■						1	[19]
32	■			■						■		26	[31]
